# Supplementary material for: MetaRibo-Seq measures translation in microbiomes
Source: Nat Commun. 2020 Jun 29;11:3268. doi: 10.1038/s41467-020-17081-z (PMC7324362; doi:10.1038/s41467-020-17081-z)
Supplement: Supplementary file 10 — Supplementary Data 7 [file 41467_2020_17081_MOESM10_ESM.zip › File2/Confidence_High_Taxonomy/426107_out.krona.html]

Javascript must be enabled to view this page.

members
magnitude
magnitudeUnassigned
count
unassigned
taxon
rank

426107\_out

29

2
29
superkingdom

1239
29
phylum

29
186801
class

186802
29
order

family
29
186803

29
841
genus

1263104
29

SRS013940\_contig\_number\_12073SRS013951\_contig\_number\_26132SRS014855\_contig\_number\_15528SRS014923\_contig\_number\_contig-100\_238.224066SRS015190\_contig\_number\_14529SRS015854\_contig\_number\_19822SRS020869\_contig\_number\_32819SRS043001\_contig\_number\_10361SRS043411\_contig\_number\_12116SRS045739\_contig\_number\_8915SRS050925\_contig\_number\_30256SRS052027\_contig\_number\_17058SRS054956\_contig\_number\_15703SRS055017\_contig\_number\_5638SRS056273\_contig\_number\_27063SRS065504\_contig\_number\_10603SRS075716\_contig\_number\_contig-100\_595.35651SRS104084\_contig\_number\_10440SRS1055034\_contig\_number\_11962SRS142503\_contig\_number\_contig-100\_209.340715SRS142781\_contig\_number\_contig-100\_855.81696SRS143070\_contig\_number\_15876SRS143598\_contig\_number\_23263SRS144362\_contig\_number\_32900SRS147919\_contig\_number\_13343SRS148091\_contig\_number\_3996SRS148159\_contig\_number\_49948SRS148424\_contig\_number\_3750SRS149879\_contig\_number\_25515
species
